# Effects of ionizing radiation on the viability and proliferative behavior of the human glioblastoma T98G cell line

**DOI:** 10.1186/s13104-018-3438-y

**Published:** 2018-05-21

**Authors:** Hossam Murad, Yaman Alghamian, Abdulmunim Aljapawe, Ammar Madania

**Affiliations:** 1 Human Genetics Division, Department of Molecular Biology & Biotechnology, Atomic Energy Commission of Syria (AECS), P.O. Box 6091, Damascus, Syria; 2 Department of Animal Biology, Faculty of Sciences, Damascus University, Damascus, Syria; 3 Department of Radiation Medicine, Atomic Energy Commission of Syria (AECS), Damascus, Syria

**Keywords:** Glioma, T98G cell line, Ionizing radiation, Radioresistance, MTT, Flow cytometry, Apoptosis

## Abstract

**Objective:**

Radiotherapy is the traditional therapy for glioma patients. Glioma has poor response to ionizing radiation (IR). Studying radiation-induced cell death can help in understanding the cellular mechanisms underlying its radioresistance. T98G cell line was irradiated with Co^60^ source by 2 or 10 Gy. MTT assay was used to calculate the surviving fraction. Cell viability, cell cycle distribution and apoptosis assays were conducted by flow cytometry for irradiated and control cells for the 10 Gy dose.

**Results:**

The SF2 value for irradiated cells was 0.8. Cell viability was decreased from 93.29 to 73.61%, while, the Sub G0/G1 phase fraction was significantly increased at 10 Gy after 48 h. On the other hand, there was an increase in the percentage of apoptotic cells which reached 40.16% after 72 h at the same dose, while, it did not exceeds 2% for non-irradiated cells. Our results showed that, the T98G cells is radioresistant to IR up to 10 Gy. Effects of irradiation on the viability of T98G cells were relatively mild, since entering apoptosis was delayed for about 3 days after irradiation.

## Introduction

Glioblastomas are tumors affecting the central nervous system. They rank among the most common primary tumors worldwide [1]. Only a small percentage of patients live for 2 years after disease onset [2]. These tumors are traditionally treated by surgery, however, it is impossible to completely eradicate the tumor due to its penetrating nature with neighboring tissues [3]. Therefore, surgery is followed by radiation therapy (60 Gy total dose within 6 weeks), which increases the chance of recovery. In most cases, radiation resistant cancer cells settle in sensitive locations of the brain, which cannot be accessed by surgery, leading to relapse [4–6].

Several studies investigated cellular behavior after exposure to IR, the capacity of radiation to stop proliferation of cancer cells and to induce apoptosis [7–9]. IR induces DNA damage, this damage can influence cell fate [10, 11]. After DNA damage occurs, DNA damage pathways are activated, leading to cell cycle arrest, DNA damage repair, cell proliferation, senescence, or apoptosis [11, 12]. Exposure to IR causes failure in cell division and loss of some genetic material, showing an aberrant distribution of chromosomes during division [13]. Previous studies showed that, preferential activation of the DNA damage checkpoint and enhanced DNA repair capacity in gliomas lead to radioresistance [9, 14, 15]. Strategies depending on targeting DNA damage response network in gliomas were applied to sensitize tumors and reverse radioresistance [16, 17]. Furthermore, another studies showed abrogating the cell cycle checkpoint could increase radiosensitivity in glioma cell line [17, 18].

Several studies showed that IR causes glioma cells to enter apoptosis [19, 20]. Apoptosis is an active mechanism of cell death, requiring the activation of a cascade of caspases genes, leading to DNA fragmentation and plasma membrane disintegration [21]. It has been shown that the extent of this process determines cells sensitivity to IR [13]. Some studies have shown that radiation did not cause apoptosis in the U343 cell line derived from glioblastoma multiforms, which makes it radioresistant [22, 23]. Full understanding of the glioblastomas response to IR and detailed radioresistance analysis may help to identify radiosensitizing agents of this fatal disease.

Several methods have been developed to measure cell sensitivity to radiation, such as the MTT spectrophotometric assay [24, 25]. The SF2 value (surviving fraction at 2 Gy) is used as an indicator for cell sensitivity to radiation. Cells are considered radioresistant if the SF2 value exceeds 0.5 and radiosensitive if the SF2 value is less than 0.5 [26, 27].

Flow cytometry has been used to determine cell viability and rate of cell death caused by radiation in cell lines derived from glioblastomas [28, 29]. Flow cytometry can also be used to study cell proliferation and cell cycle distribution utilizing cellular DNA content histograms. Annexin-V assay is a quantitative method that can be used to detect and quantify cells entering apoptosis [30, 31].

The aim of this study was to estimate the sensitivity of the T98G cells (as a model of glioblastomas) to IR and to investigate the effects of a high dose of IR on the T98G cell lines at the cellular levels, by studying the cell viability, cell cycle and apoptosis to investigate the behavior of this cells to this dose.

## Main text

### Materials and methods

#### Cell culture

T98G cells were kindly provided by Prof. Dr. P. Bécuwe, University of Nancy. Sub cultures of this cell line were maintained in complete RPMI-1640 medium containing 10% FBS (fetal bovine serum), 2 mM glutamine, 0.1 mg/ml each of penicillin and streptomycin, at 37 °C and 5% CO_2_. All cell culture media were purchased from (Gibco, USA).

#### Cells irradiation

Cultures of T98G cells were irradiated in the irradiation reference laboratory at (AECS) using a Co^60^ source (Theratron 80, USA), Doses used were 2 or 10 Gy and the dose rate was of 447 mGy/min.

#### MTT spectrophotometric assay

The MTT kit (Roche, Germany) was used as previously reported [32] to measure T98G cells sensitivity to radiation. Cells were cultured in 96-well plates (5000 cells/well) and irradiated with the 2 Gy. The value of SF2, which reflects the sensitivity of the cells to radiation, was calculated using the following equation: SF = 2 − (t delay/t doubling time), where: “t delay” is the time needed for irradiated cells to reach an absorbance equal to that of control cells, “t doubling time” is the time cells need to double their number.

#### Cell cycle analysis

T98G cells were treated with a 10 Gy of IR and returned to the incubator for 6, 24, 48 and 72 h. Cells were collected, washed with PBS and stained for cell cycle analysis using BD Biosciences cell cycle test kit (BD Biosciences, USA). For each sample, 1 × 10^4^ cells were analyzed using the BD FACSCalibur flow cytometer (Becton–Dickinson, USA).

#### Cell viability assay

T98G cells were irradiated with the 10 Gy, then, harvested after 6, 24, 48 and 72 h. Cells then washed with PBS buffer and stained for cell viability using BD Biosciences viability kit.

#### Apoptosis assay

10 Gy irradiated T98G cells were collected after 6, 24, 48 and 72 h of irradiation. Cells then washed with PBS buffer and stained using BD Biosciences Apoptosis Detection Kit I.

#### Statistical analysis

The SPSS 23 software (SPSS, Chicago) was used in statistical analysis. The Student-*t* test was applied to analyze the differences between treatments. Differences were considered statistically significant at *P < 0.05.

## Results

### Radiosensitivity and viability of T98G cells

**Fig. 1:**
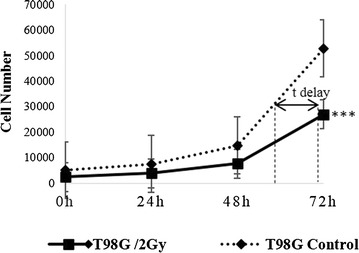
Determination of the radiosensitivity of the T98G cell line using the MTT method. Absorbance values were converted to cells number using a logarithmic line equation of a stander curve for each point, Y axis: cell number, X axis: time. Irradiation of T98G cells with a 2 Gy dose caused a growth delay of about 12 h compared to non-irradiated cells (control). The experiment has been repeated three times and data are expressed as the mean ± SD

**Fig. 2:**
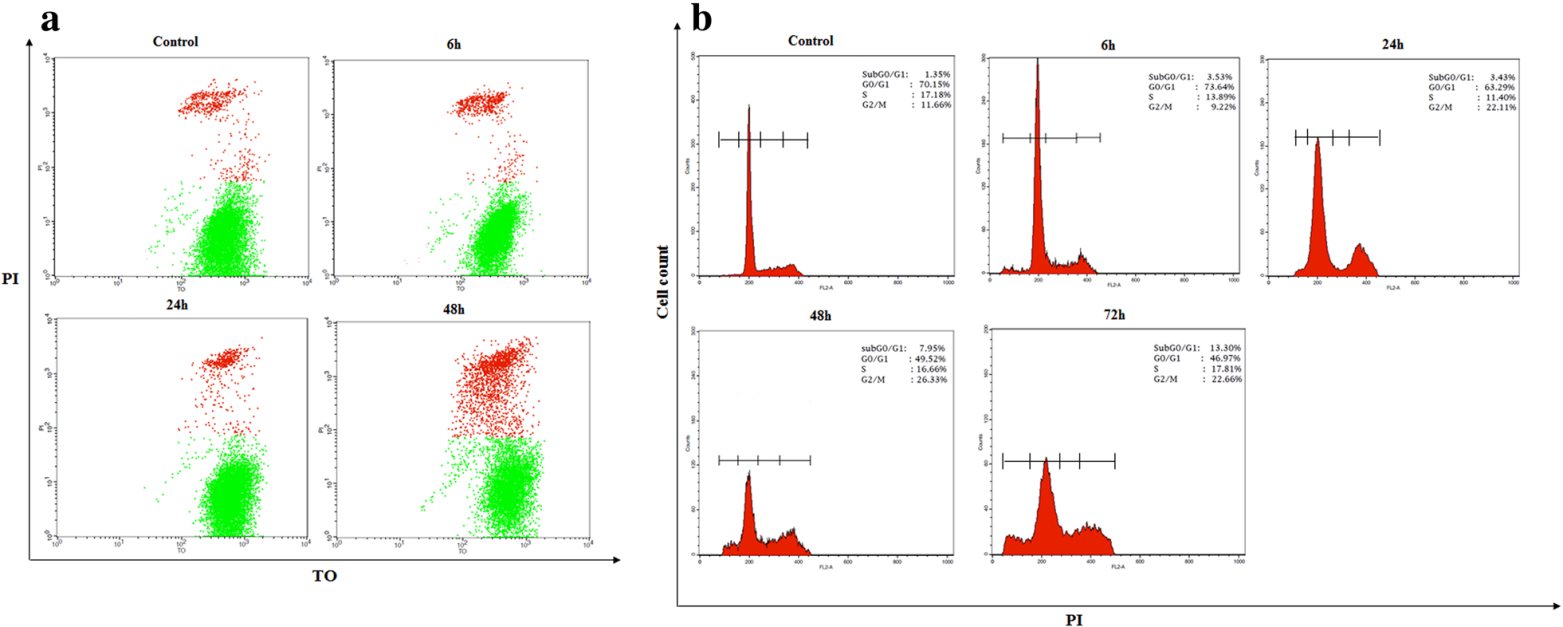
**a** Effect of irradiation with a 10 Gy dose on the viability of T98G cell line. Flow cytometry histogram showing the changes in percentage of dead (colored by PI, in red) and live cells (colored by TO and PI, in green), with elapsed time after irradiation indicated. **b** Effect of irradiation with a 10 Gy dose on T98G cell cycle distribution. Flow cytometry histogram showing the cell distribution according to DNA content

The SF2 value for cells irradiated with 2 Gy was 0.8, which is clearly greater than 0.5, indicating that the T98G cells are radioresistant. As shown in (Fig. 1), growth of irradiated cells was delayed about 12 h compared to non-irradiated cells. Viability of T98G cells exposed to a 10 Gy was dropped to 93.29, 91.62 and 73.61% after 6, 24 and 48 h respectively, (Fig. 2a).

### Effect of IR on the cell cycle of T98G cells

As shown in Fig. 2b, the percentage of dead cells increased to 3.53, 3.43, 7.93 and 13.3% after 6, 24, 48 and 72 h of irradiation respectively. We found that the percentage of cells found in G1 phase was decreased after 6, 24, 48 and 72 h to 73.64, 63.29, 49.52 and 46.97% respectively, after irradiation with 10 Gy. While the percentage of 10 Gy irradiated cells found in G2 phase was 9.22, 22.11, 26.33 and 22.66% after 6, 24, 48 and 72 h respectively showing a slight G2/M cell cycle arrest.

### Effect of IR on apoptosis of T98G cell line

**Fig. 3:**
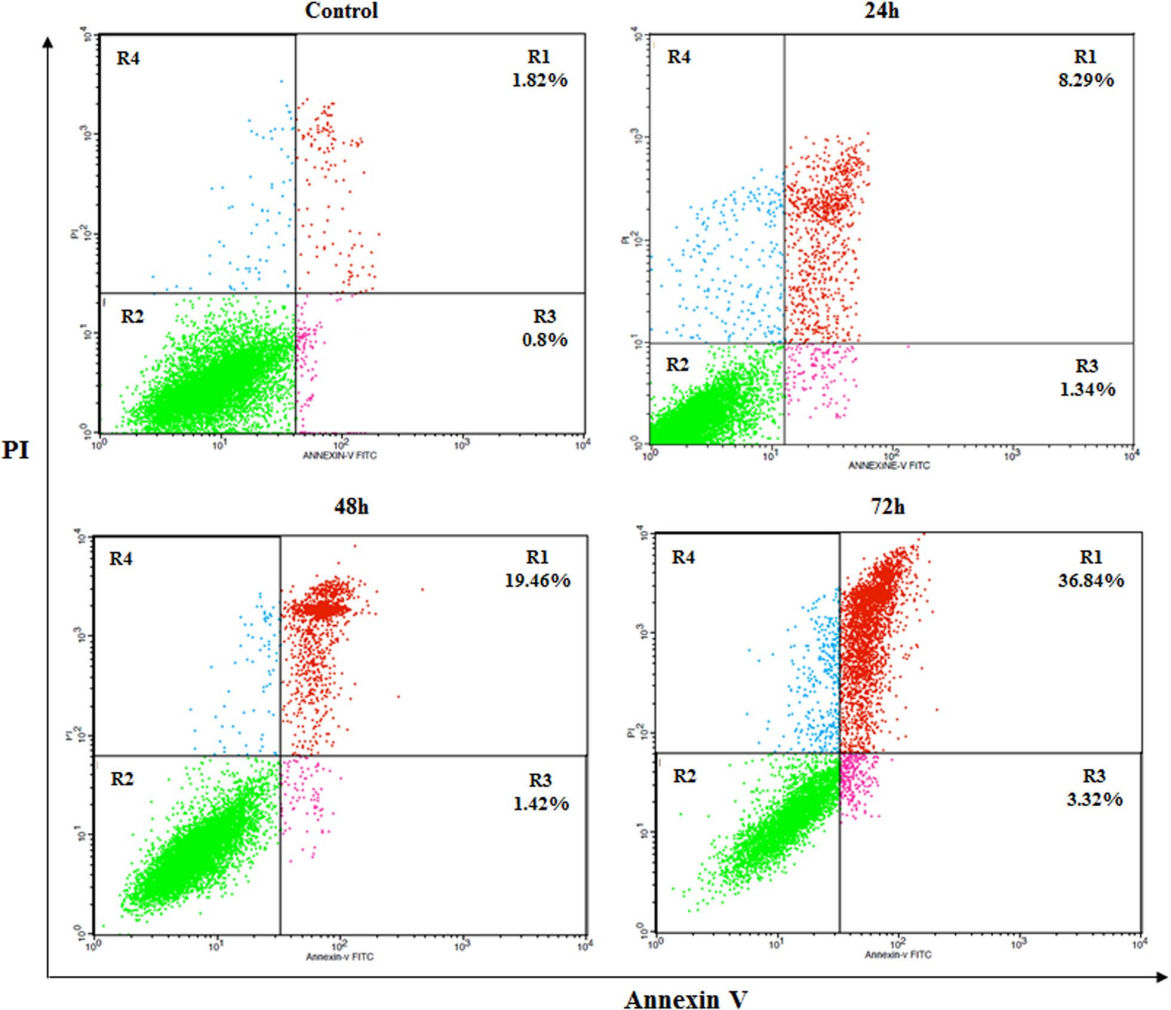
Effect of irradiation with a 10 Gy dose in inducing apoptosis in the T98G cell line. Shown is the percentage of early apoptosis cells (annexin V^+^ PI^–^, R3 quadrant) and late apoptosis cells (annexin V^+^ PI^+^, R1 quadrant) at 24, 48, 72 h after irradiation

We used the double staining method (annexin V-FITC and IP) and flow cytometry to determine the percentage of cells undergoing programmed cell death due to irradiation. As shown in Fig. 3, we distinguished four groups of cells: live (annexin V^−^ PI^−^, R2 quadrant), early apoptotic (annexin V^+^ PI^−^, R3 quadrant), late apoptotic (annexinV^+^ PI^+^, R1 quadrant) and necrotic (annexin V^−^ PI^+^, R4 quadrant). Flow cytometric analysis demonstrated that after irradiation with 10 Gy, apoptosis rate (sum of the R1 and R3 quadrants) increased from 9.63 to 20.88% and to 40.16% after 24, 48 and 72 h respectively.

## Discussion

Glioblastomas represent one of the deadliest cancer types, where affected patients generally die within 2 years after disease onset [33]. In spite of the high radioresistance of glioblastoma cells, IR remains one of the traditional therapies for those tumors [34, 35]. Radioresistance of cancer cells was the subject of numerous studies, due to its importance in cancer therapy practice and implications in several molecular pathways, such as DNA repair, cell cycle check points and cell death [14, 36, 37]. The high resistance of glioblastoma cells to radiotherapy is attributed to weak entrance into programmed cell death induced by IR [38]. Ionizing radiation induces damage to the genetic material of the cell, negatively affecting several vital cellular mechanisms [14]. As a response to these damages, cells can select one of several possible pathways according to the nature, intensity and duration of the induced effect (chemotherapy, radiotherapy, pharmacological drugs, etc.) [11, 12]. Cells can continue their division, ignoring the induced effect, or their cell cycle arrest until damages are repaired, then enter into senescence, differentiate, or enter into apoptosis if the damages are irreparable [3]. Furthermore, the fate of the tumor cell after radiotherapy is determined by its characteristics, such as type and tissue of origin [39]. The SF2 value is considered as an indicator for cells radiosensitivity [26, 27]. Our results showed that, SF2 value was 0.8, so the T98G cells were considered as radioresistant (Fig. 1). Previous study by Liu et al. showed that, T98G cells had SF2 value reach to 0.7 compared to other human cell lines [40]. In our study, the viability of irradiated cells (up to 10 Gy) was slightly decreased after 48 h (Fig. 2a). Furthermore, exposing T98G cells to a 10 Gy, (which is considered relatively a high dose), induced a significant cell death only after 72 h (Fig. 2b). Roy et al. showed that, the sensitivity of the tow U118 and U87 cells (glioblastoma cell lines) to IR at 6 Gy was more than (> 2-fold) compared to T98G cells [41]. Another study by Yao et al. revealed that, there was a significant inhibition of cell proliferation at 20 Gy for (GB-1, T98G, U251-MG, and U373-MG) cells at 24 h, and the apoptosis did not occur in any these cells following irradiation [38]. Our results showed, no accumulation of irradiated T98G cells in the SubG0/G1 phase after a 10 Gy at 6, 24 and 48 h, whereas, we observed a limited cell death at 72 h. Yao et al. did not observe any dead T98G cells irradiated at 5 Gy in the SubG0 phase after 4 days [38]. Our results showed that, a 10 Gy irradiation resulted in a G2/M cell cycle arrest. This phase gives the cells an opportunity to repair damaged DNA induced by irradiation. However, when DNA damage is irreparable cells undergo apoptosis [9, 42, 43].

In addition, double staining with annexin-V-FITC and PI showed that, the rate of apoptotic irradiated T98G cells reached 40.16% at 72 h. Ma et al. pointed that, there was only 16% of death rate after 96 h for U251 MG cells (glioblastoma cells) irradiated at 7 Gy [9]. Several studies suggested a combination treatment for T98G cells to improve their radiosensitivity to IR. Tani et al. showed that, the continuous down-regulation of γ-glutamylcysteine synthetase (γ-GCS) expression by hammerhead ribozyme (as a potential anticancer gene therapy), increased the cytotoxicity of the T98G cells to IR [44]. Although, treatment of T98G cells with BI 2536 (as an inhibitor for Polo-like kinase 1) caused mitotic arrest and increased apoptosis in irradiated cells after 24 h [45].

## Conclusions

Taken together, our results showed that even in the high-dose, 10 Gy, cells did not respond to ionizing radiation after 24 h, while, these cells needed about 72 h to inter in apoptotic phase by a rate not exceeding 40% at the same dose. We conclude that, the ionizing radiotherapy alone even in a high dose, does not lead to the efficient treatment in advanced grade glioblastoma patients.

## Limitations

Further studies are necessary in order to elucidate the molecular mechanisms causing cell resistance to IR, and to identify the genes responsible for radioresistance. These genes would be potential targets for targeted therapies that would improve response to IR and force cancer cells to enter cell death.

## Abbreviations

SF2: surviving fraction at 2 Gy
MTT: (3-(4,5-dimethylthiazolyl-2)-2,5-diphenyltetrazolium bromide)
TO: thiazole orange
PI: propidium iodide
